# The Influence of the Washing Process on the Impedance of Textronic Radio Frequency Identification Transponder Antennas

**DOI:** 10.3390/ma16134639

**Published:** 2023-06-27

**Authors:** Magdalena Nizioł, Piotr Jankowski-Mihułowicz, Mariusz Węglarski

**Affiliations:** 1 Department of Metrology and Diagnostic Systems, Rzeszów University of Technology, Wincentego Pola 2, 35-959 Rzeszów, Poland; 2 Department of Electronic and Telecommunications Systems, Rzeszów University of Technology, Wincentego Pola 2, 35-959 Rzeszów, Poland

**Keywords:** RFID textronic transponder, textronics, textile antennas, wearable antennas, washable electronics

## Abstract

Antennas dedicated to RFID systems created on textile substrates should maintain strictly defined parameters. During washing, the materials from which such antennas are made are exposed to mechanical and chemical exposure—degradation of the parameters characterizing those materials may occur, which in turn may lead to a change in the parameters of the antenna. For research purposes, four groups of model dipole antennas (sewn with two types of conductive threads on two fabrics) were created and then they were subjected to several washing processes. After each stage of the experiment, the impedance parameters of the demonstration antennas were measured using indirect measurements. Based on the obtained results, it was found that these parameters change their values during washing, and that this is influenced by a number of factors, e.g., shrinkage of the substrate fabric.

## 1. Introduction

### 1.1. Textiles in Electronics

Progress in various fields of technology and its interdisciplinary uses cause an increase in the popularity and accessibility of intelligent technologies. In recent years, smart wearable devices used, among others, in the medical [1,2,3,4,5,6], sports [7,8,9,10] or military and emergency services [11,12] sectors have become extremely popular. In order to meet the growing requirements for the comfort of using such devices, designers’ attention has been turned towards intelligent textiles and electronic textiles (e-textiles) [13,14]. The latter turn out to be particularly interesting because they have new functionalities based on the electronic elements [13,15,16] implemented in their structure.

Modern electronic textiles are based on the integration of classic fabrics with conductive materials—most often foils, threads, yarns or conductive polymers—and their production does not differ from standard methods used in the textile industry, such as printing, weaving, embroidery or sewing, during which non-conductive fibers are replaced. It is possible to create conductive paths, diodes or detectors of chemical or physical parameters on the fabrics constituting the substrate [13,16,17,18].

Electronic textiles have found particular application in communication systems, where they are used to create various types of antennas [19,20]. The geometry of the antenna is created using conductive material on a textile dielectric substrate. As a rule, such an antenna is integrated with a piece of everyday or protective clothing, due to which it must meet certain conditions. One of them is to make the antenna in a way that does not spoil the aesthetics of the product with which it is integrated. Equally important, if not the most important aspect, is the fact that such an antenna must have a certain level of resistance to washing.

When washing classic textiles, negative effects may occur as a result of temperature, detergent or mechanical factors (friction, spinning)—this can be a change in the appearance of the material (change in shade or texture) or a change in its size (shrinkage, stretching). In the case of e-textiles, there is also the issue of changing the electrical parameters of the conductive material, which in turn may disrupt or even prevent the operation of the entire system of which they are a part [21,22].

### 1.2. UHF RFID Transponder

The possibility of creating antennas on textile substrates has opened the prospect of developing radio frequency identification (RFID) devices, especially in the area concentrated around the human body. The use of textile antennas in RFID transponders enables the permanent integration of such a device with textile industry products, thanks to which it is possible to construct a system that operates throughout the life cycle of a specific product.

Also, the widespread availability of portable electronic devices supports the rapid spread of RFID systems in the market, as it is extremely easy for such devices to connect with each other to obtain available information.

The RFID system for the UHF (ultra-high-frequency) band typically operates in the 860–960 MHz range. The determination of the appropriate frequency value depends on the region of the world in accordance with the regulations enforced in a given area.

**Figure 1:**
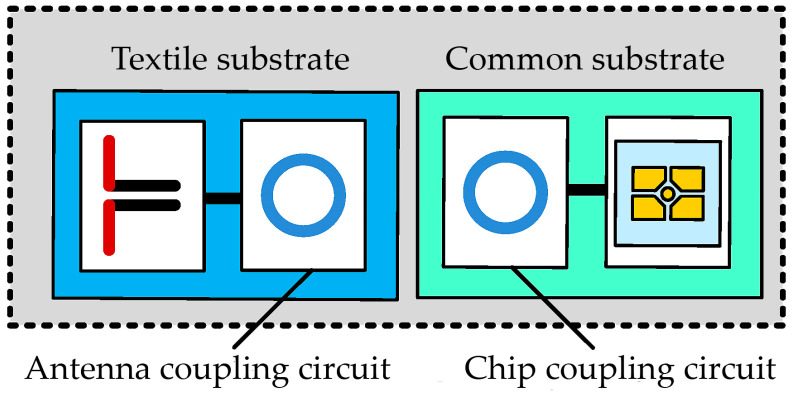
Block diagram of textronic RFID transponder (RFIDtex tag).

Passive solutions account for a large share of the market for RFID devices, as the lack of batteries allows for significant cost reductions. A typical passive RFID tag consists of a chip and an antenna—in the vast majority of cases, both of these elements are permanently connected to each other. The patent PL 231291 B1 “Textronic RFID transponder” by Jankowski-Mihułowicz P., Węglarski M. [23] presents a concept based on the galvanic separation of both elements by means of a special coupling circuit (Figure 1).

Both the chip and the antenna have a coupling circuit in the form of properly profiled loops, between which there is mutual induction. Its value is sufficient to ensure proper impedance matching of the chip and the antenna.

The presented structure enables the aesthetic integration of the textile antenna with the target product while making a microelectronic system in the form of, for example, a button. Not only does the lack of a physical connection of both elements increase the reliability of the transponder, it also prevents a number of problems from occurring at the production stage.

In the scientific literature, a lot of attention is devoted to RFID transponders for the UHF band, and the directions of research are constantly being developed—solutions based on e-textiles are becoming more and more popular [24,25,26,27,28]. As mentioned in Section 1.1, textile transponders (and especially their antennas, which are usually a textile element) must be resistant to the same exposures as the textile products with which they are integrated. On account of that, aspects such as the impact of moisture or washing on the parameters and operation of transponders [29,30,31,32,33] are tested.

The main purpose of this publication Is to present the effect of the washing process on the impedance of the textile RFID tag antenna for the UHF band. The study of the impact of washing on tags with textile antennas has already been a subject of research, but in the available literature only results regarding its impact on the read range (main effect) [29,34] can be found. The authors decided to delve deeper into this topic and investigate the impedance of the tag antennas (the cause of that effect), which affects that read range.

## 2. Materials and Methods

### 2.1. Textile Antenna

The idea and problems arising during the design of an antenna for a textile RFID tag are described in detail in publications [23,35].

**Figure 2:**
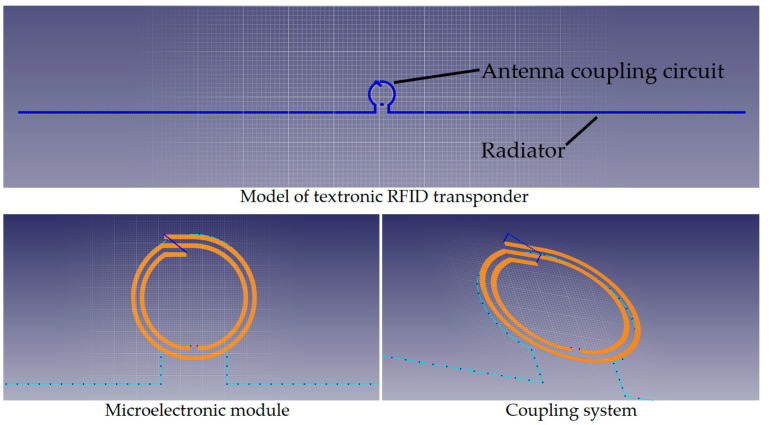
Model of the antenna with microelectronic module.

In the case of this publication, the object of research is a simple dipole with a length shorter than half the wavelength (16 cm) with a coupling circuit in the form of a loop with a diameter of 5.6 mm. Figure 2 shows the design of the transponder built on the patent “Textronic RFID transponder”, made in the EMCoS Studio 2021 program.

For the transponder to work properly, the antenna must be impedance-matched to the chip and the quality of this match is determined by the power transfer coefficient (Equation (1)) [23,36].

(1) τ=4Re⁡zTARe⁡zTCRe⁡zTA+zTC2+Im⁡zTA+zTC2

According to the design assumptions, the real part of the antenna impedance should be equal to several ohms, while the imaginary part to several hundred. These values are determined by the chip parameters, because in the case of a perfect matching both impedances are conjugate (*Z_TA_* = Z_TC_*).

Antenna modules (radiator with a coupling circuit) were constructed with two types of conductive threads—litz wire PACKLitzWire 10 × 0.04 mm (Rudolf Pack GmbH & Co. KG, Gummersbach, Germany) and Syscom Agsis (Syscom Technology Inc., Columbus, OH, USA) (nylon thread covered with a layer of silver, characterized by its resistance to washing). The samples were embroidered on two types of fabrics, the composition of which is shown in Table 1.

The WL4715 (Tkaniny24, Iława, Poland) material is a cotton fabric with a linen structure. The presence of lycra in its composition provides slight elasticity. WL4948 (Tkaniny24, Iława, Poland) is a denim fabric with high abrasion resistance.

Used substrate materials were selected on the basis of experience from previous research on textronic RFID tags described in other authors’ publications [35,37]. The research that is the subject of this article is a continuation of research on antennas for which a high convergence of results with the theoretical model has been determined.

**Figure 3:**
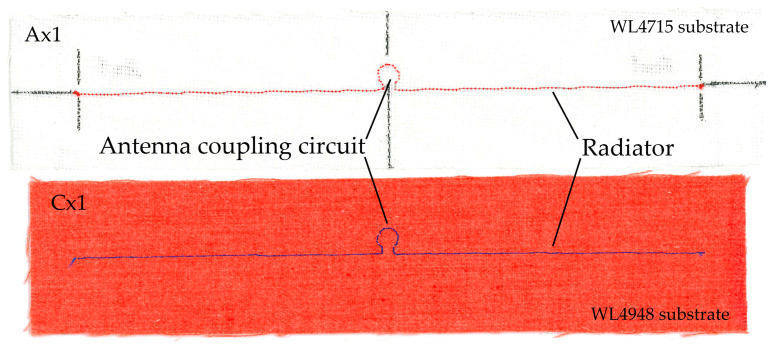
The RFIDtex transponder examples.

The samples were embroidered on an embroidery machine, in which the conductive thread is the bobbin thread and the upper thread is ordinary polyester thread. The appearance of exemplary samples is shown in Figure 3.

Created for the research were

- Ten samples sewn on WL4715 fabric with Agsis thread (group A, marked as Ax1–Ax10);
- Eleven samples sewn on WL4715 with litz wire (group B, marked as Bx1–Bx11);
- Five samples sewn on WL4948 with Agsis thread (group C, marked as Cx1–Cx5);
- Five samples sewn on WL4948 with litz wire (group D, marked as Dx1–Dx5).

The varying number of samples between the four groups is due to the different availabilities of the materials they are composed of.

The electronic module was created on a flexible substrate made of DuPont Pyralux copper-clad laminated composite (constructed of kapton polyimide film) LF9150R (DuPont de Nemours, Inc., Wilmington, DE, USA). The dielectric layer of this laminate was covered with a layer of copper with a thickness of 35 µm.

### 2.2. Measurement Procedure

When measuring the parameters of RFID transponders, the measurement methods used in the case of classic radio systems cannot be used, because the impedance of the chip and the antenna is expressed in complex form (Equation (2)) [36].

(2) $$Z_{TA}=R_{TA}+jX_{TA}$$

where *R_TA_*—antenna resistance; *X_TA_*—antenna reactance (inductive).

In addition, the value of the chip’s impedance changes depending on the amount of power supplied to it from the antenna and the antenna’s impedance is susceptible to changes due to environmental conditions. Furthermore, there are other problems, e.g., with the connection of measuring equipment (no compatible connectors in transponders [36]).

The publication in [36] presents in detail the procedure for measuring the impedance of the RFID tag antenna operating in the HF and UHF bands. After adapting to the available conditions, it is widely used in both scientific and industrial centers.

In order to determine the impedance of the transponder antenna, indirect measurements should be made using a passive differential probe with signal-to-signal contact tips and two 50 Ω ports of a vector network analyzer (VNA). The measurement system is a linear two-port network, in which pairs of terminals are the antenna power terminals and virtual ground.

The measurement procedure should be started with the calibration of the measuring chain. A dedicated calibration substrate with short-, open- and matched-load standards can be used for this purpose. The probe tips were attached to the appropriate elements on the substrate and the relevant parameters were measured and corrected using the VNA.

The probe tips were connected to the terminals of the coupling circuit of the microelectronic system, and then the parameters of the scattering matrix (**S**) were measured using the VNA.

However, this matrix does not contain direct information about the sought antenna impedance, so it should be determined as the differential impedance between the two ports of the analyzer (Equation (3)) [36].

(3) $$Z_{TA}=\frac{U_{1}-U_{2}}{I_{0}}$$

After introducing the voltages *U*_1_ and *U*_2_ to the parameters of the impedance matrix, the equation takes the form of (4) [36].

(4) $$Z_{TA}=Z_{11}-Z_{12}-Z_{21}+Z_{22}$$

After taking into account the relationship between the **Z** and **S** matrices, the equation describing the measured antenna impedance takes the form of (5) [36].

(5) $$Z_{TA}=\frac{2Z_{0}\left(S_{12}S_{21}-S_{11}S_{22}-S_{12}-S_{21}+1\right)}{\left(1-S_{11}\right)\left(1-S_{22}\right)-S_{12}S_{21}}$$

where *Z*_0_ = 50 Ω.

In order to facilitate and accelerate the measurement data acquisition process, the EMCoS SimDAT tool was used to determine the antenna impedance, which is used to process and analyze simulation data; however, it is possible to import external data to it and this option was used. After importing the measurement data in the form of **S** parameters, it was possible to automatically convert it to the **Z** matrix and select the desired parameter.

## 3. Results

### 3.1. Impedance Measurements of Model Antenna

**Figure 4:**
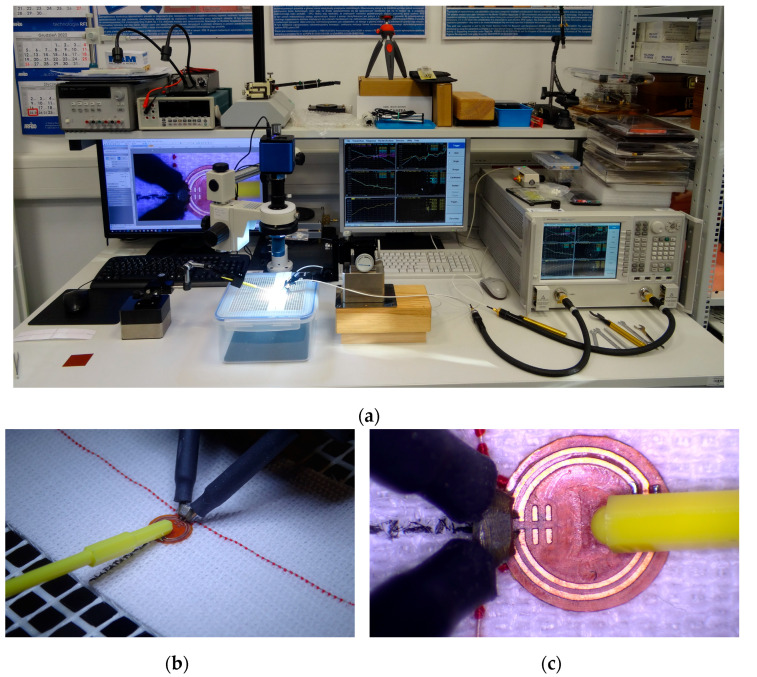
Laboratory research stand: (**a**) photo of the setup; (**b**) closeup of antenna under test; (**c**) microscopic view of the microelectronic module with attached probe.

In accordance with the measurement procedure provided in Section 2, the impedance measurements of model antenna modules in the frequency range from 0.5 to 1.2 GHz were taken. Figure 4 shows the view of the laboratory stand used during the tests.

The station is equipped with a Keysight PNA-X N5242A vector network analyzer with a special PacketMicro DPSS201505 SS05–0053 probe. The acquisition of measurement data was performed using a PC. The orientation of the microelectronic system on the antenna and the attachment of the probe to the microelectronic module were controlled using a microscope.

**Figure 5:**
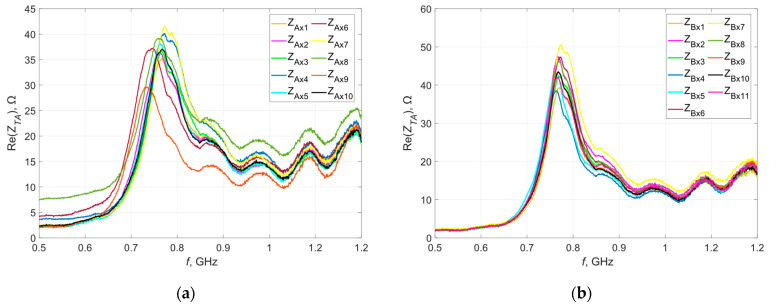

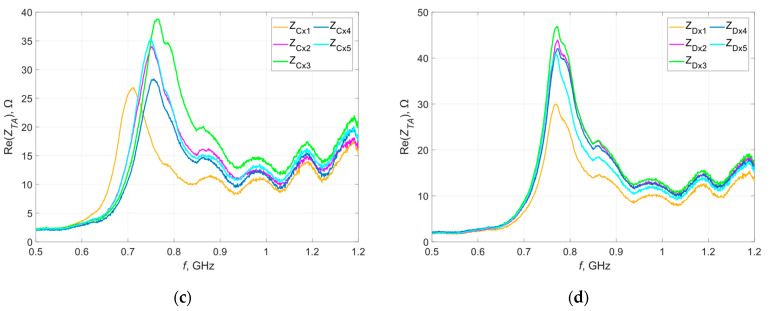
Waveforms of the real part of the impedance of the model antennas from group (**a**) A; (**b**) B; (**c**) C; (**d**) D.

**Figure 6:**
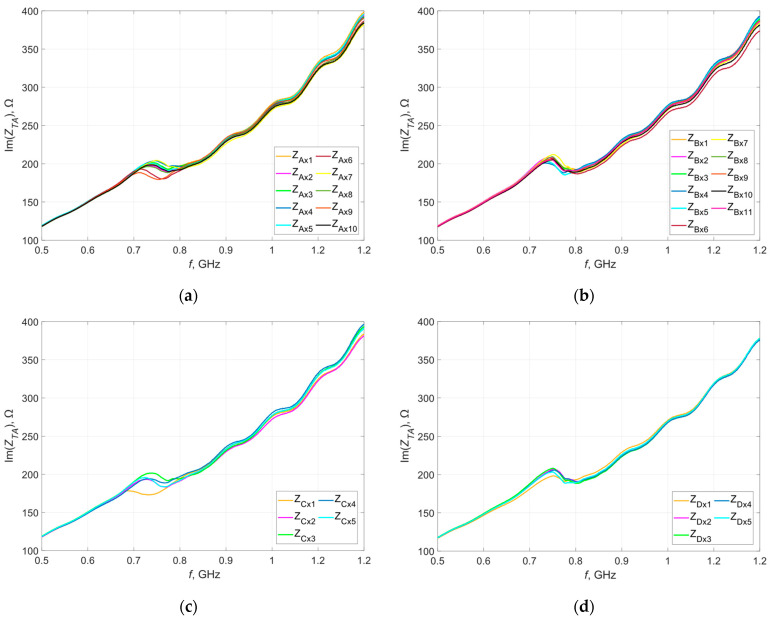
Waveforms of the imaginary part of the impedance of the model antennas from group (**a**) A; (**b**) B; (**c**) C; (**d**) D.

Figure 5 and Figure 6 show the waveforms of the real and imaginary parts of the impedance as a function of frequency. Measurements were taken before washing.

Within individual groups (A, B, C, D), there is a certain dispersion of impedance values, especially in the real part. This can be due to either the production process (the embroiderer stretches the thread, which may cause a difference in geometric dimensions in relation to the design) or the measurement procedure carried out, or both. While the elements of the measurement path are taken into account during the calibration of the measurement system, there is some randomness in the position of the microelectronic system on the antenna.

The creation of model antenna systems with machines guarantees an appropriate level of repeatability of the production process. The samples were treated as a statistical sample, and the obtained results were averaged. Sufficiently good metrological parameters of the apparatus used at the measuring station enable the analysis of the dispersion of the collected data by means of statistical analysis, taking into account only random errors, such as, for example, an inaccurate position of the microelectronic system on the antenna. This dispersion is characterized by the sample standard deviation (*s*), which is defined by Equation (6).

(6) $$s=\sqrt{\frac{\sum {\left(Z_{i}-Z_{av}\right)}^{2}}{n-1}}$$

where *Z_i_*—sample impedance value for a specific frequency, *Z_av_*—average impedance value in a given group of samples for a specific frequency, *n*—number of samples in a group.

**Figure 7:**
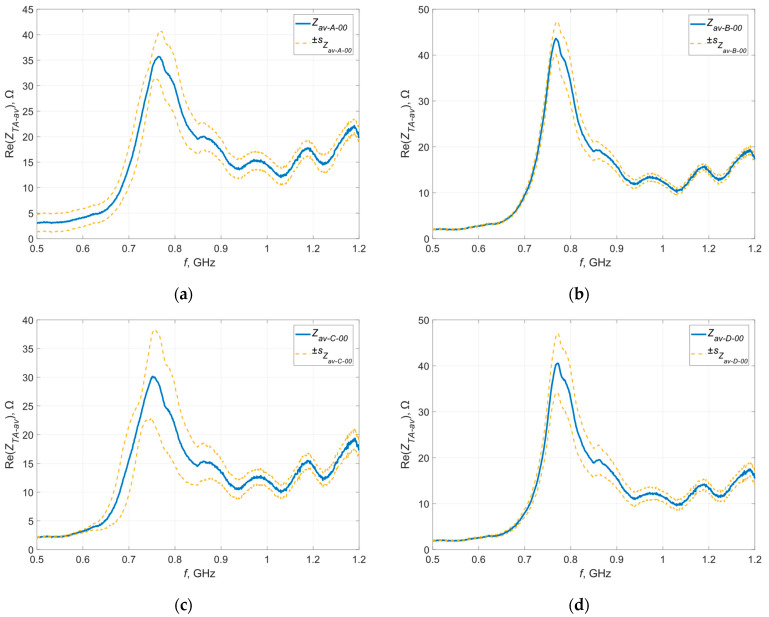
Standard deviation of the real part of the impedance of the model antennas from group (**a**) A; (**b**) B; (**c**) C; (**d**) D.

**Figure 8:**
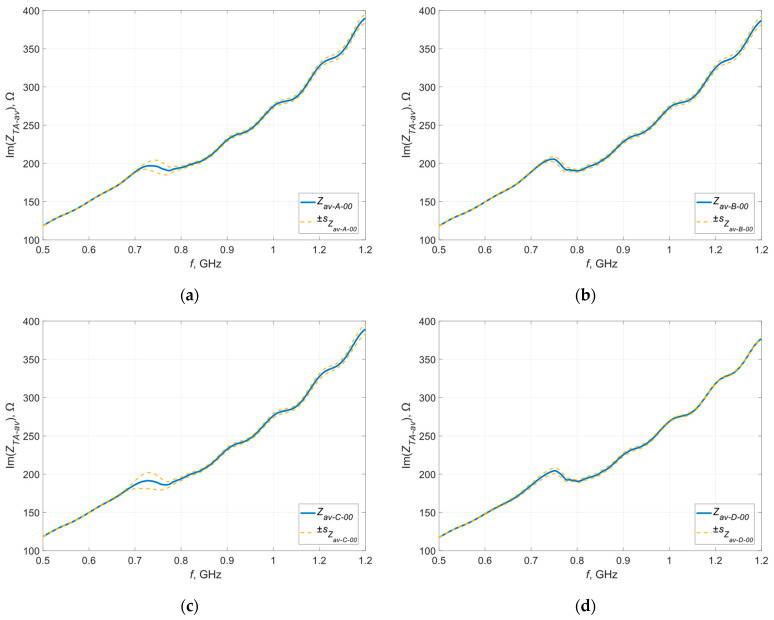
Standard deviation of the imaginary part of the impedance of the model antennas from group (**a**) A; (**b**) B; (**c**) C; (**d**) D.

The deviation was calculated for each frequency at which the measurement was taken and calculations were performed for the real and imaginary parts of the impedance—the results obtained in graphical form are presented in Figure 7 and Figure 8.

The orange curves represent the standard deviations determined at each measurement point, defining the dispersion of the measured values around the mean at a given point. The largest dispersion of measured values is observed near the resonance frequency, which is a direct result of the characteristics of the occurring phenomenon. In resonance, even a small change in the parameters in the tested circuit can cause significant deviations in the measured impedance. In the case of samples sewn with Agsis thread, a greater dispersion is observed than in the case of samples sewn with the litz wire. This is caused by the characteristics of the materials from which the threads are made and their electrical parameters. The influence of the skin effect, which is very limited in the case of the litz wire, also cannot be ruled out.

**Figure 9:**
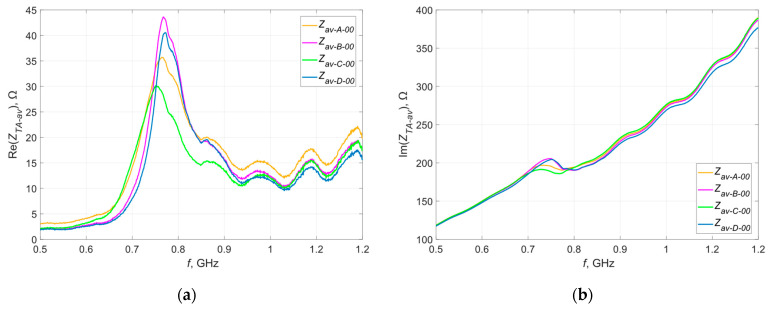
Waveforms of the (**a**) real part; (**b**) imaginary part of the impedance of the model antennas from all groups.

Before proceeding to the next stage of research, the average waveforms of the real and imaginary impedances from all analyzed groups were compared (Figure 9).

Such a juxtaposition makes it possible to assess the credibility of the results obtained in the subsequent stages of the research, because it can be seen that the substrate parameters do not affect or have a relatively small impact on both parts of the impedance waveforms. The same conclusions are presented in publication [35], where the influence of textile substrates on the performance of textronic RFID tags was thoroughly investigated. The obtained values, however, strongly depend on the parameters of the thread that the antenna is made of (the similarity of the samples from groups A and C, as well as B and D, being most visible in the waveforms of the imaginary part).

### 3.2. The Effect of Washing on the Impedance of Model Antennas

As presented in Section 3.1, the impedance values of the model antennas were taken as reference values and the study of the impact of the repeated washing process on the obtained results was started. The samples from all groups were washed in an automatic washing machine with the following washing program settings: cotton, temperature 30 °C, washing time 1 h, spinning 1100 rpm. This program has been selected based on the most common recommendations of manufacturers of textile products, which are placed on their labels. The detergent was also used in recommended amounts.

**Figure 10:**
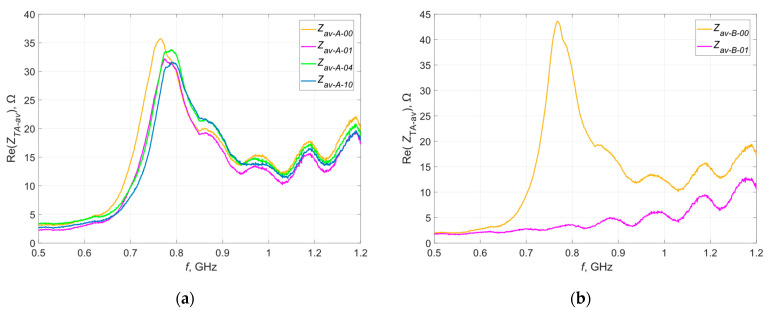

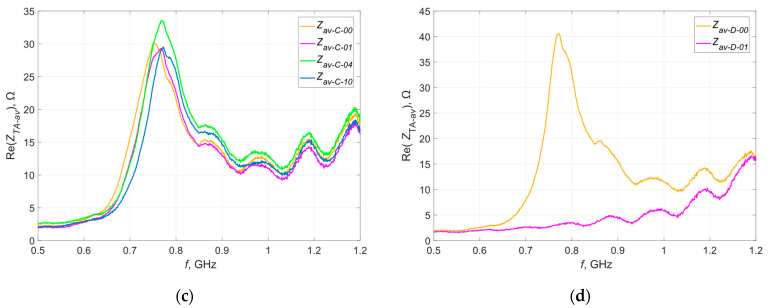
Waveforms of the real part of the impedance of the model antennas after recurrent washing from group (**a**) A; (**b**) B; (**c**) C; (**d**) D.

**Figure 11:**
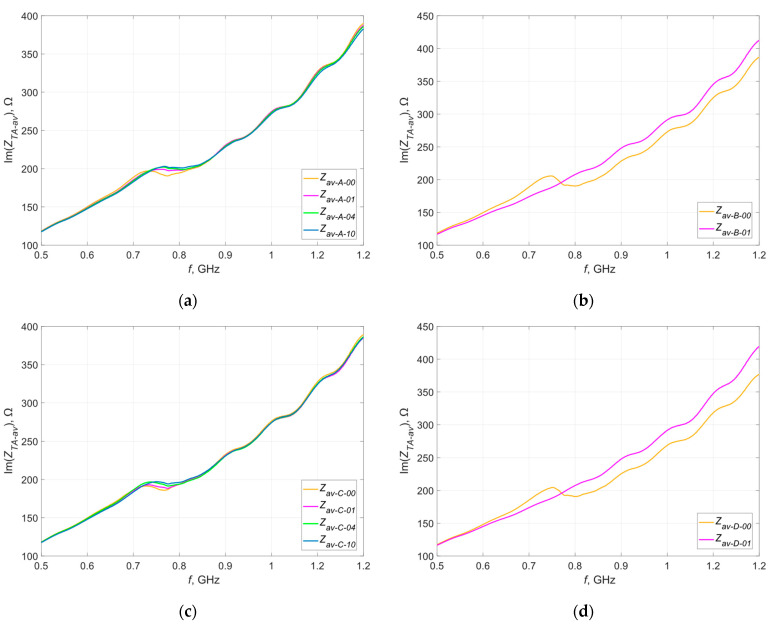
Waveforms of the imaginary part of the impedance of the model antennas after recurrent washing from group (**a**) A; (**b**) B; (**c**) C; (**d**) D.

After 1, 4 and 10 washing cycles, the impedance of all samples was tested again. Same as before, the results obtained within each group were averaged—the resulting waveforms of the real and imaginary parts of the impedances in relation to the reference waveforms (before washing) are shown in Figure 10 and Figure 11.

The letter A, B, C or D in the curve description indicates the group of samples for which the results are presented. Numbers 00, 01, 04 and 10 indicate the number of washing cycles performed—successively before washing, after 1 wash, after 4 washes and after 10 washes.

**Figure 12:**
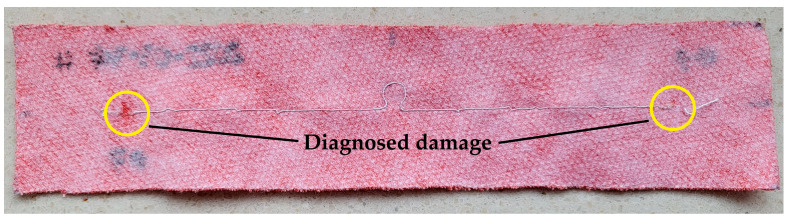
Example of a damaged sample.

The testing of samples from groups B and D was completed after the first wash. The analysis of the obtained characteristics clearly indicates damage to the antennas (lack of resonance on the plotted curve)—the litz wire with which they were sewn was broken, often in many places (yellow markings in Figure 12). This was to be expected, because litz wire is not intended for applications subjected to this type of stress. These damages are most likely due to the mechanical stress caused by the rotating drum of the washing machine [38].

The waveforms of the real and imaginary impedances of the samples embroidered with Syscom Agsis thread changed their shapes with successive washing cycles—the value of the resonant frequency successively increased, which suggests a change in the geometric dimensions of the tested antennas. Changes in the maximum values of both components near resonance frequencies were also noted—this may be the result of changes in the resistance of the conductive thread. However, the changes in resistance are different for different threads. According to the documentation of the Agsis thread manufacturer, this thread is characterized by an approximately constant resistance value for the analyzed number of washing cycles. This is suggested by the results presented in publication [39], in which the authors examined the effect of washing on the resistance of conductive threads.

**Figure 13:**
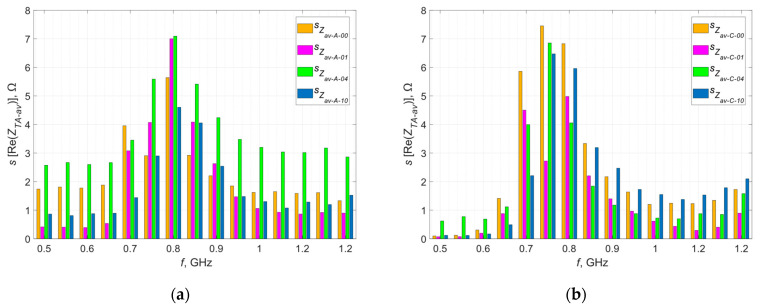
Standard deviation of the real part of the impedance of the model antennas at selected points of frequency from group (**a**) A; (**b**) B.

**Figure 14:**
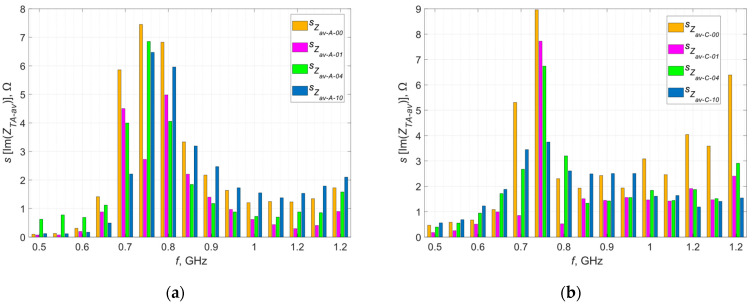
Standard deviation of the imaginary part of the impedance of the model antennas at selected points of frequency from group (**a**) A; (**b**) B.

For samples from groups A and C, the dispersion of both impedance components around the averaged values was determined. The values of standard deviations at selected points (calculated after every stage of the experiment) are presented graphically in Figure 13 and Figure 14.

The presented graphs do not show unequivocal increases in the dispersion of the measured values after successive washing cycles. On this basis, it can be concluded that subsequent washing cycles do not cause uncontrolled changes in the measured parameters within a given group at individual stages of the experiment. The reported dispersion of values results rather from random errors occurring during the measurements.

**Figure 15:**
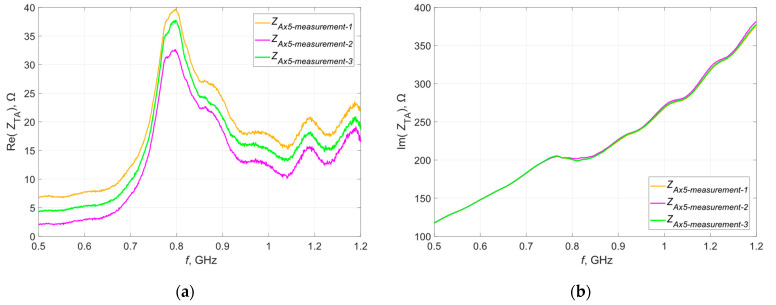
Waveforms of the (**a**) real; (**b**) imaginary part of the impedance of the selected antenna.

**Figure 16:**
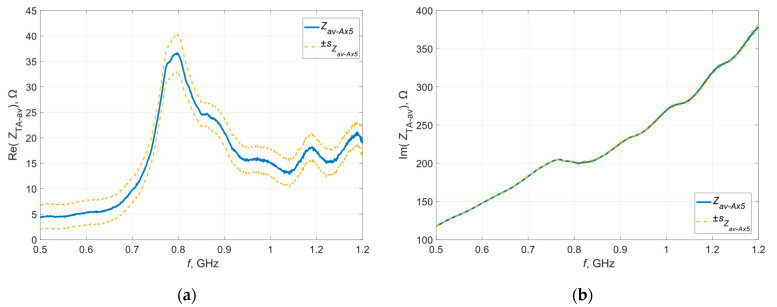
Standard deviation of the (**a**) real; (**b**) imaginary part of the impedance of the selected antenna.

To confirm these assumptions, a random sample was selected and measurements of its impedance were taken three times. Figure 15 shows the waveforms obtained during each measurement. The obtained characteristics were averaged and, similarly to the previous stages of experiments, the dispersion of the measured values around the average was determined—the obtained results are presented in graphical form in Figure 16.

The results obtained during repeated testing of the same sample using the same measurement method and the same apparatus and under constant conditions are characterized by the repeatability of the shape of the resulting curve as a function of frequency, but there is still a scatter of values. The only element that changed during this experiment was the position of the microelectronic circuit on the tested antennas—this clearly suggests a significant impact of this factor on the obtained results.

For control purposes, after successive washing cycles, measurements of the length of the tested samples were taken, as the geometric dimensions of the antenna have a significant impact on the impedance parameters of the antenna. The measurements were taken digitally using image analysis for this purpose—determining the length of the scanned samples after subsequent washes and comparing them to the pattern (scanned sample before washing). The results for the samples are shown in Table 2.

It was observed that each tested sample successively shortened, which is the direct cause of the change in the frequency of the antenna’s self-resonance—the shorter the antenna, the higher the value of the resonant frequency. The largest recorded changes in the length concerned the radiators of the tested antennas, but a change in the geometric dimensions of the coupling circuit was also observed. This affects the quality of the antenna coupling with the microelectronic system and, consequently, the values of the tested parameters.

## 4. Discussion

The analyzed solution of the textronic RFID transponder for the UHF band is already a useful construction, which is confirmed by the publications of this research team including the co-authors of this article. However, this topic is still being developed and further aspects that may have a measurable impact on improving the project are being investigated.

The production of a textronic RFID tag for the UHF band requires consideration of many aspects regarding both the electronic and textile layers. Before integrating this element, e.g., with clothing, the transponder’s resistance to the same factors that affect the product to which it will be connected has to be considered.

This article analyzes the effect of the washing process on the impedance of the textronic transponder antenna, which is one of the key parameters, because the correct operation of the transponder requires proper impedance matching of the antenna and the chip. Four groups of samples sewn with two types of conductive threads on two base fabrics were prepared for the tests.

First of all, based on the obtained results, it should be stated that not every conductive thread is suitable for creating textile antennas subjected to the washing process. All samples sewn with the litz wire were destroyed after the first wash—the breakage of the model antennas in many places suggests mechanical damage resulting from the rotation of the washing machine drum.

The second thread used—Syscom Agsis—in accordance with the manufacturer’s declaration, is resistant to washing. All the samples sewn with this thread were not mechanically damaged; therefore, it was possible to analyze the changes occurring in the waveforms of the real and imaginary parts of the antenna impedance. The obtained data show that the value of the self-resonance frequency of all samples changed with successive washing cycles, which is caused by a change in the geometric dimensions of the tested antennas and this change is mainly due to the shrinkage of the substrate.

Impedance values within individual groups of samples are characterized by a certain dispersion. Based on the research carried out so far, it can be concluded that they are more the effect of random errors occurring during the measurements than of the degradation of, e.g., the thread parameters. These errors may result from the imperfect location of the microelectronic circuit above the coupling circuit of the antenna or appear already at the stage of sample production.

The results obtained so far lead to the conclusion that the washing process significantly affects the impedance components of the textronic RFID tag antenna. This impact may change with an increase in the number of washing cycles performed, which is a further stage of future research. It is also worth considering the aspects of reducing or completely eliminating this impact, e.g., by securing antennas with protective materials.

The quality of the coupling of the antenna with the microelectronic circuit also has a significant impact on the obtained results; therefore, during the measurements, special attention should be paid to the exact position of this system over the coupling loop in the antenna. This can be achieved by automating this process.

## Figures and Tables

**Table 1:** Basic specifications of the used fabrics.

	WL4715	WL4948
Composition	cotton 97% spandex 3%	cotton 52% polyester 48%
GSM (grams per square meter) range	230	260

**Table 2:** Measurements of geometric dimensions of the model antennas.

Sample	Total Length/Loop Diameter before Washing in cm	Total Length/Loop Diameter after 1 Washing in cm	Total Length/Loop Diameter after 4 Washings in cm	Total Length/Loop Diameter after 10 Washings in cm
Ax1	16.03/0.54	15.73/0.51	15.67/0.51	15.58/0.51
Ax2	16.00/0.53	15.71/0.51	15.62/0.51	15.57/0.50
Ax3	16.00/0.53	15.71/0.51	15.63/0.50	15.56/0.50
Cx1	16.04/0.54	15.75/0.52	15.69/0.52	15.60/0.51
Cx2	15.99/0.55	15.73/0.53	15.64/0.52	15.61/0.52
Cx3	16.01/0.57	15.75/0.56	15.64/0.56	15.59/0.55

## Data Availability

All calculated and measured data will be provided upon request to the correspondent authors by email with appropriate justification.
